# Guselkumab, a Novel Monoclonal Antibody Inhibitor of the p19 Subunit of IL-23, for Psoriatic Arthritis and Plaque Psoriasis: A Review of Its Mechanism, Use, and Clinical Effectiveness

**DOI:** 10.7759/cureus.51405

**Published:** 2023-12-31

**Authors:** Christian K Kerut, Maxwell J Wagner, Charles P Daniel, Claire Fisher, Emmilee J Henderson, Caroline R Burroughs, Sam Amarasinghe, Olga Willett, Shahab Ahmadzadeh, Giustino Varrassi, Sahar Shekoohi, Alan D Kaye

**Affiliations:** 1 School of Medicine, Louisiana State University Health Sciences Center at New Orleans, New Orleans, USA; 2 School of Medicine, Louisiana State University Health Sciences Center, Shreveport, USA; 3 Department of Anesthesiology, Louisiana State University Health Sciences Center, Shreveport, USA; 4 Pain Medicine, Paolo Procacci Foundation, Rome, ITA

**Keywords:** autoimmune inflammatory diseases, monoclonal antibody therapy, plaque psoriasis, psoriatic arthritis, guselkumab

## Abstract

Psoriatic arthritis and plaque psoriasis are autoimmune conditions affecting multiple organs, including the skin. The pathophysiology and etiology of these conditions are not fully understood; however, numerous factors are believed to play a critical role, including genetics and environmental risk factors. Furthermore, research suggests the IL-23/IL-17 pathway partially mediates these diseases. Once the IL-23 receptor is bound and activated, two subunits, p19, and p40, act through different signaling pathways. Ultimately, inflammation is produced through the effector molecule, IL-17, other cytokines, and tumor necrosis factor (TNF). Traditionally, these chronic conditions have been treated with TNF-α inhibitors and methotrexate, a dihydrofolate reductase inhibitor. Although successful in inhibiting the immune system, these drugs can have many adverse effects due to their broad targets. In recent years, more targeted therapy has become popular. Guselkumab is a monoclonal antibody that inhibits the p19 subunit of IL-23. It has been FDA-approved to treat both plaque psoriasis and psoriatic arthritis. Clinical trials showing guselkumab’s efficacy have been promising, even showing improvement in symptoms of plaque psoriasis patients resistant to adalimumab, a TNF-α inhibitor. Guselkumab has also been shown to be well tolerated with a similar safety profile as other biologics inhibiting the immune system. In addition to its efficacy in treating plaque psoriasis and psoriatic arthritis, the mechanism of action offers a targeted approach that may minimize the broad immunosuppressive effects often associated with traditional therapies, providing a potential advantage in the long-term management of these autoimmune conditions.

## Introduction and background

Since the first FDA approval of monoclonal antibodies in 1986, the development and use of biologics for previously difficult-to-treat diseases have grown enormously. Related to high specificity and low side effect profiles, biologics have become among the highest-selling and most popular drug classes [1]. Developed by Janssen Pharmaceuticals, Beerse, Belgium, guselkumab is a fully-humanized monoclonal antibody that targets the pro-inflammatory cytokine IL-23 [1,2]. In 2017, guselkumab received FDA approval for treating moderate-to-severe plaque psoriasis, making it the first selective IL-23 inhibitor with this approval [3]. In 2020, guselkumab was granted FDA approval for active psoriatic arthritis (PsA) [4].

Plaque psoriasis is an autoimmune disorder that primarily affects the skin, causing hyperproliferation of keratinocytes, manifesting as well-defined erythematous plaques covered with silvery-white scales [5]. PsA, on the other hand, is a type of spondyloarthritis often preceded and accompanied by psoriasis [6]. PsA and plaque psoriasis are immune-mediated disorders where an overactive immune system triggers inflammation and abnormal cell growth [7]. Among the many cytokines contributing to these processes, IL-23 plays a crucial role. By targeting and inhibiting IL-23, guselkumab helps regulate the immune system, reduces the number of pro-inflammatory cytokines, and mitigates the immune response. Unlike traditional treatment options that primarily target cytokines like tumor necrosis factor-alpha (TNF-α), this review examines studies comparing guselkumab with these older treatment options. In this regard, guselkumab has significantly reduced the symptoms and inflammatory markers of patients with these autoimmune disorders. Safety-wise, guselkumab has shown similar results to other biologics and is tolerated well. Due to its significant effectiveness and safety profile, guselkumab is a monoclonal antibody that can be used to treat plaque psoriasis and PsA, especially in those who have not responded adequately to conventional therapies. This review focuses on various aspects of guselkumab, including mechanism of action, pharmacology, efficacy, adverse effects, comparativeness to other treatments, and usage in treating PsA and plaque psoriasis.

## Review

Pharmacodynamics and pharmacokinetics of guselkumab

Guselkumab is a human monoclonal antibody that selectively binds to and inhibits the p19 subunit of IL-23 cytokine [2,8]. It is an immunoglobulin gamma 1 lambda monoclonal antibody [9]. IL-23 plays a pivotal role in the development of psoriasis and PsA, with guselkumab acting by blocking this cytokine before it can activate Th17 helper T-cells, which are key mediators of tissue damage [10]. Without activation by IL-23, the Th17 cells cannot produce IL-17, a cytokine detected in excess in the blood of patients with psoriasis and PsA [11]. The inflammatory properties of IL-23 and IL-17 have been shown to play a role in both of these disease processes [12].

In clinical practice, guselkumab is administered by subcutaneous injection [13]. Based on data gathered from clinical trials, the approved dosing of guselkumab is a 100mg dose given at the start of treatment, then another 100mg dose at week four, and then 100mg given every eight weeks for the duration of treatment [13]. The pharmacokinetic models of guselkumab give insight into the body’s metabolism of the drug. The half-life has been measured at 18.1 days [9]. This slow rate of removal from the body provides insight into the lengthy dosing regimen of guselkumab. Clearance of the drug had a median measurement of 0.516 L/day, and the distribution volume was 13.5 L [9]. These measurements were subject to significant variation depending on patient-specific variables such as race, weight, or patients’ diabetic status [9]. For example, in a diabetic patient, the clearance rose to 0.578 L/day, and in patients whose weights were in the first quintile, the clearance dropped to 0.443 L/day [9]. These variations are important clinical considerations when prescribing guselkumab and reinforce the necessity of monitoring patients taking the drug.

Like other immunosuppressive drugs, guselkumab has led to slightly increased infection rates among treated patients [14]. Upper respiratory tract infections were the most common infections in the treated patients [15]. At the 24-week mark, infection rates were only 2% higher than those of the placebo group and were observed mainly in groups receiving monthly dosages, deviating from the approved eight-week dosing regimen [14]. When the infection rates were re-analyzed at 112 weeks, they measured below those seen in patients receiving placebo treatment [15]. In less than 3% of patients, there was evidence of an injection site reaction [15]. Elevated liver alanine aminotransferase and aspartate aminotransferase levels were seen in approximately 5% of patients; however, most of these patients did not need to discontinue treatment [15]. About 4% of patients receiving treatment with guselkumab saw mild decreases in neutrophil count; however, these decreases were transient except for one patient who had a grade 4 neutrophil decrease [15]. At 112 weeks, over 730 patients had been treated with guselkumab, and there were single-digit numbers of patients who had serious reactions, including major adverse cardiovascular events (n=3), opportunistic infection (n=3), and suicidal ideation (n=3) [15]. There was only one patient who died during the two-year study, and that was due to an automobile accident [15].

Guselkumab use in PsA

*PsA* *pathology and pathophysiology*

PsA is a spondyloarthritis defined by musculoskeletal manifestations of chronic inflammatory arthritis affecting 20-30% of people with psoriasis [16,17]. The pathophysiology of PsA suggests genetic risk factors predisposing patients to a chronic inflammatory process triggered by mechanical or environmental stress [18]. Enthesitis, the most prominent pathologic lesion in PsA, is inflammation at the attachment site of ligaments, tendons, and joint capsules to the bone. This chronic inflammatory disease also manifests with peripheral arthritis, dactylitis, and axial disease. The ClASsification for Psoriatic ARthritis (CASPAR) criteria, the most accepted clinically diagnostic criteria for PsA, assigns a point value to various manifestations of inflammatory arthritis, including skin psoriasis, nail lesions, dactylitis, rheumatoid factor, and juxta-articular bone formation [18]. PsA may develop into irreversible joint damage characterized by bone erosion in about 50% of patients, significantly impacting the quality of life [19]. While the pathology of both psoriasis and PsA is not yet fully understood, research suggests a contributory mechanism mediated by the IL-23/IL-17 pathway, a mediator of host defense at mucosal barriers. IL-23 is a widely expressed regulator of cytokine release and tissue inflammation throughout the body. When IL-23 binds with its tissue receptor, IL-23’s two subunits, p19 and p40, act through Janus kinase 2 and TYK2 signaling pathways, respectively. Once this IL-23 regulator-receptor complex is activated, the IL-23 signaling pathway produces inflammation via the production of its effector molecule, IL-17, and various other cytokines, chemokines, and TNF (16). With the IL-23/IL-17 mechanism well defined in the pathophysiology of cutaneous psoriasis, mouse studies have shown that IL-23 receptors are expressed by T-cells at the location of tendon insertion into bone. These IL-23 receptors show the production of IL-17 and inflammation with the experimental administration of IL-23 at this site, suggesting a similar pathologic mechanism of PsA in humans [20].

*Guselkumab mechanism of action in PsA* *and plaque psoriasis*

Guselkumab, an FDA-approved treatment for both PsA and psoriasis, is a human monoclonal antibody that inhibits IL-23 by selectively binding to the p19 subunit [21]. Although not completely understood, the guselkumab antibody binds with high affinity and specificity to the IL-23 p19 subunit, thereby preventing the interaction of the IL-23 cytokine with its cellular tissue receptor. Guselkumab-mediated inactivation of the IL-23 cytokine prevents activation of the IL-23 receptor, blocking the IL-23 signaling pathway and subsequent release of proinflammatory cytokines that lead to both PsA and plaque psoriasis [22]. These proinflammatory cytokines often upregulate genes involved in psoriasis and lead to keratinocyte hyperproliferation [22]. Guselkumab and other p19 selective monoclonal antibodies offer high specificity compared to the alternative monoclonal antibody treatment options.

Comparison of Guselkumab to Alternative Monoclonal Antibodies

Alternative monoclonal antibody treatment options for PsA target a variety of targets, including IL-12/23, IL-17, and IL-23, all of which mediate the chronic inflammation of PsA [23]. Although these biologics have unclear benefits to costs, it is clear that PsA and plaque psoriasis decrease patients' quality of life [23]. Ustekinumab, an IL-23/IL-12 monoclonal antibody, targets the p40 subunit expressed by both IL-23 and IL-12 [16]. While Ustekinumab proved effective in the treatment of psoriatic disease, p19 selective monoclonal antibodies outperformed their p40 counterparts in clinical trials, suggesting a protective effect of uninhibited IL-12 in psoriatic disease (Table 1) [24].

**Table 1:** Comparison of phase 3 randomized controlled clinical trials of licensed monoclonal antibodies for the treatment of psoriatic arthritis. All studies are evaluated by the percentage of patients achieving the American College of Rheumatology 20 response rates (ACR20) after 24 weeks of intervention [25].

Citation	Groups studied and intervention	Results and findings	Conclusions
[25]	Guselkumab DISCOVER-1: 381 patients with active psoriatic arthritis were randomly assigned to groups to be treated with 100 mg of guselkumab in intervals of four weeks, eight weeks, or treatment with a placebo.	59% of the group treated every four weeks with 100 mg of guselkumab, 54% of the group treated every eight weeks with 100 mg of guselkumab, and 22% of the group given placebo met the ACR20 standard at the 24-week endpoint.	59% of patients treated with the optimal dosing of guselkumab met the ACR20 standard for improvement by week 24.
[21]	Guselkumab DISCOVER-2: 741 patients with active psoriatic arthritis were randomly assigned to groups to be treated with 100 mg subcutaneous injections of guselkumab in intervals of four weeks or eight weeks or treatment with a placebo.	64% of the group treated every four weeks with 100 mg of guselkumab, 64% of the group treated every eight weeks with 100mg of guselkumab, and 33% of the group given placebo achieved the ACR20 standard at the 24-week endpoint.	64% of patients treated with the optimal dosing of guselkumab met the ACR20 standard for improvement by week 24.
[26]	Secukinumab FUTURE-3: 414 patients with active psoriatic arthritis were randomly assigned to groups to be treated with 300 mg of secukinumab or 150 mg of secukinumab or a placebo at weekly intervals for weeks one to four, followed by every four weeks thereafter.	48.2% of the group treated with 300mg of secukinumab, 48.2% of patients treated with 150mg of secukinumab, and 16.1% of those given the placebo achieved the ARC20 standard at the 24-week endpoint.	48.2% of patients treated with the optimal dosing of secukinumab met the ARC20 standard for improvement by week 24.
[27]	Risankizumab KEEPsAKE-1: 964 patients with active psoriatic arthritis were randomly assigned to groups to be treated with 150 mg of Risankizumab at weeks zero, four, and 16 or treatment with a placebo.	57.3% of the group treated with 150 mg of risankizumab and 33.5% of the group given placebo achieved the ARC20 standard at the 24-week endpoint.	57.3% of patients treated with the optimal dosing of risankizumab met the ARC20 standard for improvement by week 24.
[28]	Risankizumab KEEPsAKE-2: 444 patients with active psoriatic arthritis were randomly assigned to groups to be treated with 150 mg of Risankizumab at weeks zero, four, and 16 or treatment with a placebo.	51.3% of the group treated with 150 mg of risankizumab and 26.5% of the group given placebo achieved the ARC20 standard at the 24-week endpoint.	51.3% of patients treated with the optimal dosing of risankizumab met the ARC20 standard for improvement by week 24.
[29]	Ustekinumab PSUMMIT-1: 615 patients with active psoriatic arthritis were randomly assigned to groups to be treated with 45 mg of ustekinumab, 90 mg of ustekinumab, or placebo at weeks zero, four, and every 12 weeks thereafter.	42.4% of the group treated with 45 mg of ustekinumab, 49.5% of the group treated with 90 mg of ustekinumab, and 22.8% of the group given placebo achieved the ARC20 standard at the 24-week endpoint.	49.5% of patients treated with the optimal dosing of ustekinumab met the ARC20 standard for improvement by week 24.
[30]	Ustekinumab PSUMMIT-2: 312 patients with active psoriatic arthritis were randomly assigned to groups to be treated with 40 mg of ustekinumab, 90 mg of ustekinumab, or a placebo at intervals of weeks zero and four. Followed by administration at week 16 for the placebo group or q12 for the treatment group.	43.8% (combined treatment groups) of the ustekinumab-treated groups and 20.2% of the group given placebo achieved the ARC20 standard at the 24-week endpoint.	43.8% of patients treated with the optimal dosing of ustekinumab met the ARC20 standard for improvement by week 24.

Secukinumab, an IL-17 monoclonal antibody, selectively targets and binds cytokine IL-17A. IL-17 is the effector in the IL-23/IL-17 signaling pathway and mediates biological functions, including joint inflammation, damage, and remodeling. Secukinumab’s neutralization of IL-17 restricts its effector activity and downstream proinflammatory effects in psoriatic arthritis [19]. While secukinumab has undergone various clinical trials, the FUTURE-3 trial is used for comparison in Table 1 due to its method comparing the approved clinical doses, 150 and 300 mg, at a 24-week endpoint evaluation of patients meeting the ACR20 criteria [26]. Lastly, guselkumab and risankizumab are selective monoclonal antibodies targeting the IL-23 p19 subunit. While the ACR20 response rates in Table 1 cannot be compared directly due to each being derived from different studies, both phase 3 trials of guselkumab evidence significant efficacy with 59% and 64% of the optimally dosed treatment group meeting the ACR20 response criteria by week 24 [21,25].

Guselkumab use in plaque psoriasis 

Among the various subtypes of psoriasis, chronic plaque psoriasis is the most common presentation, with over 80% of psoriatic patients exhibiting this complaint. Other subtypes of psoriasis skin manifestations include guttate psoriasis, inverse psoriasis, pustular psoriasis, and erythrodermic psoriasis. With the WHO issuing a report in 2016 describing the prevalence of psoriasis in adults in the United States (US) as approximately 3.2%, this disease is a significant concern in the medical field [31]. The typical age of onset has a bimodal distribution at the age of 16-22 years and 55-60 years, and it occurs equally in men and women [32]. The characteristic skin lesions of plaque psoriasis are well-demarcated, erythematous, pruritic plaques most commonly occurring on extensor regions, the scalp, the lumbosacral region, and the umbilicus [33].

The etiology and pathophysiology of psoriasis remain complex and not yet fully understood; however, strong evidence supports a genetic predisposition to psoriasis. A study involving 61 twin pairs indicated a concordance rate approximately two to three times higher in monozygotic twins compared to dizygotic twins [34,35]. Additionally, many environmental triggers have been implicated in the pathogenesis of psoriasis, including trauma or injury, infections caused by staphylococcal and streptococcal species, and social factors such as stress, alcohol consumption, obesity, and tobacco use [36]. Though there have been limited studies on the initiation phase of psoriatic skin lesions [37-39], the maintenance phase has been well established and has thus led to the development of many effective treatments. In chronic disease, inflammatory and mature myeloid dendritic cells in the skin are activated and produce IL-23 and IL-12. IL-23 activates and drives the Th17 pathway and is required for the proliferation of IL-17-producing cells. In turn, IL-17 drives keratinocyte activation and proliferation, leading to stimulated keratinocytes producing cytokines, furthering the development of inflammatory psoriatic lesions [40].

This IL-23/IL-17 axis has been a crucial therapeutic target for plaque psoriasis. Upon binding of guselkumab to IL-23, IL-23 is inhibited from binding to its receptor on target cell surfaces. This blocks the IL-23 branch of the inflammatory process; however, the IL-12 axis is not affected by the drug. Other biologic treatments include TNF-a inhibitors, IL-17 inhibitors, anti-IL-17 receptor antibodies, non-specific inhibitors of both IL-23 and IL-12, and intracellular targets of the IL-23/IL-17 pathway. However, several studies have proposed a protective effect of IL-12, and treatments blocking the IL-12 axis may be counterproductive in the disease healing process [41,42].

Guselkumab gained approval from the US FDA in 2017 for treating moderate to severe plaque psoriasis. In 2020, it was the first IL-23 inhibitor approved to treat active PsA [4]. Clinical trials, such as VOYAGE I and VOYAGE II, have demonstrated that guselkumab is more effective than placebo and adalimumab in treating moderate to severe plaque psoriasis [43]. VOYAGE I and II trials were phase III, double-blinded placebo and active comparator-controlled trials. Though the efficacy was evaluated using several tools, this review will focus on using the Psoriasis Area and Severity Index (PASI) in the VOYAGE I and II trials.

The PASI scores range from 0-72, with a higher score representing more severe disease. The PASI score was graded based on patient improvement from their baseline; for example, a PASI 90 means a 90% reduction in disease severity. The VOYAGE I trial followed subjects through 48 weeks. VOYAGE I included three subject groups: those who received only guselkumab 100 mg; a group who received placebo at weeks zero, four, and 12 followed by guselkumab 100 mg through week 44; and those who received only adalimumab 80 mg. VOYAGE II included similar subject groups, but the study also included a crossover design. In this design, guselkumab responders with a PASI score of 90 at week 28 were randomized to receive guselkumab (maintenance group) or placebo followed by guselkumab after a loss of response (withdrawal group) [44]. The crossover study design offered many benefits: it confirmed the efficacy of data from VOYAGE I, allowed subjects to serve as their own corresponding controls, and lastly, simulated non-continuous treatment of the drug, a frequent occurrence in real clinical practice. Additionally, adalimumab non-responders (< PASI 90) switched to guselkumab treatment at week 28. This arm of the study showed promising outcomes for patients who will switch between biological treatment classes. A summary of results from VOYAGE I and II are included in Table 2.

**Table 2:** Clinical summary of results from VOYAGE I and II PASI: Psoriasis area and severity index

Trial	Group studied	Results				Conclusions
VOYAGE I [45]		PASI 90 (relative to baseline)				Guselkumab was superior to both placebo and adalimumab, with a higher percentage of patients achieving and maintaining a 90% or greater response rate.
		Week 16	Week 24		Week 48	
	Guselkumab only	73.3	80.2		76.3	
	Placebo-guselkumab crossover	2.9	--		--	
	Adalimumab only	49.7	53.0		47.9	
VOYAGE II [46]		% maintenance PASI 90 Week 48				Consistent with VOYAGE I trials, guselkumab proved superior to both placebo and adalimumab with higher percentages of patients achieving PASI 90. Furthermore, it assessed the outcomes of non-continuous treatment versus maintenance of guselkumab treatment. Lastly, it proved effective in treating adalimumab non-responders.
	Guselkumab only (maintenance group)	88.6				
	Guselkumabà Placebo (withdrawal group)	36.8				
		PASI 90 (relative to baseline)				
	Adalimumab non-responders à Guselkumab	Week 24		Week 48		
		54.8		88.1		

Discussion

Plaque psoriasis and PsA can be complex to manage because of their autoimmune pathogenicity and debilitating effects. While various treatments exist for these conditions, monoclonal antibodies are gaining popularity as therapy, particularly for refractory cases unresponsive to traditional treatment methods [1]. Guselkumab has significantly reduced symptoms and inflammatory markers of psoriatic arthritis and plaque psoriasis. The safety profile of guselkumab is like that of other biologics, with common adverse effects being upper respiratory tract infections, injection site reactions, and mild decreases in neutrophil count, but serious adverse events have been rarely observed that would discredit guselkumab [2]. However, there is still a need for future studies looking at the long-term efficacy and safety of guselkumab.

Two clinical trials demonstrated significant improvement in symptoms of PsA, demonstrating their clinical effectiveness [21,25]. In other clinical trials, selective p19 subunit inhibitors including guselkumab outperformed p40 (subunit of IL-12) inhibitors, such as ustekinumab, in the treatment of psoriatic arthritis. Ustekinumab inhibits both p19 (subunit of IL-23) and p40 (subunit of IL-12), which suggests a potential protective mechanism in preserving IL-12 [24].

Guselkumab has also been approved for the treatment of severe plaque psoriasis. Clinical trials have demonstrated that guselkumab is more effective than both placebo and adalimumab, a TNF-a inhibitor, in treating moderate to severe plaque psoriasis (43). Furthermore, when patients who failed to respond to adalimumab were switched to guselkumab, symptoms were significantly reduced. Responses were measured and compared using the PASI. This improvement when switching to guselkumab demonstrates the potential clinical effectiveness while avoiding other biologics. Limitations of this review are that there are limited studies on guselkumab, especially comparing it to other biologics for plaque psoriasis and PsA. Due to its novelty, guselkumab is still a promising drug but requires further study.

## Conclusions

Guselkumab is a monoclonal antibody that targets the p19 subunit of IL-23. It is approved for the treatment of moderate-to-severe plaque psoriasis and active PsA, both of which are autoimmune disorders affecting a significant proportion of the population. Clinical trials have showcased that patients with guselkumab achieve faster onset of improvement, faster clearance of psoriasis lesions, and unparalleled safety for patients, making it a valuable consideration for both plaque psoriasis and PsA management. Compared to other biologics with different mechanisms, guselkumab performed better in reducing symptoms, highlighting the potential clinical use in the future. This study has highlighted guselkumab, its clinical use, effectiveness, and safety profile by discussing multiple clinical trials. However, due to the novelty of this drug, further research must be conducted comparing the effectiveness and safety of this drug while investigating its long-term effects.
